# Immunological Control of Polyoma Virus Oncogenesis in Mice

**DOI:** 10.1038/bjc.1973.2

**Published:** 1973-01

**Authors:** J. M. Gaugas, A. C. Allison, F. C. Chesterman, R. J. W. Rees, M. S. Hirsch

## Abstract

**Images:**


Br. J. Cancer (1973) 27, 10.

### IMMUNOLOGICAL CONTROL OF POLYOMA VIRUS ONCOGENESIS IN MICE

J. M. GAUGAS[1], A. C. ALLISON[2], F. C. CHESTERMAN[3], R. J. W. REES[1] AND M. S. HIRSCH[2]

*From the National Institute for Medical Research, Mill Hill[1]; Clinical Research Centre, Harrow[2]; Imperial Cancer Research Fund Laboratories, Mill Hill[3]*

Received 28 July 1972. Accepted 17 August 1972

**Summary.—Adult CBA mice thymectomized, treated with antilymphocytic globulin (ALG) and inoculated with human leprosy organisms were accidentally infected with polyoma virus and all developed tumours. After cessation of ALG administration, some animals were given spleen cells from syngeneic donors immunized with polyoma virus; none developed tumours. Similar results were obtained in mice deliberately infected with polyoma virus but not with leprosy organisms. Passive transfer of antibody before but not after virus inoculation prevented tumour formation in immunosuppressed recipients. Virus infection in thymectomized, lethally irradiated and bone marrow reconstituted mice resulted in only a very low incidence of tumours. These results emphasize the role of immunological surveillance in preventing polyoma tumour formation under natural conditions.**

LONG-TERM immunosuppression increases the frequency of neoplasia and reduces induction time after laboratory infection or inoculation of polyoma or leukaemogenic viruses into adult mice (Law, 1966; Allison and Taylor, 1967; Allison and Law, 1968). During an attempt to transmit progressive human leprosy (*Mycobacterium leprae*) to mice, multiple tumours arose in all mice thymectomized as adults and treated with heterologous antilymphocytic IgG (ALG), which impairs cell-mediated immunity. These tumours were indistinguishable from those induced by polyoma virus. Virological, serological and electron microscopical studies demonstrated that polyoma virus, possibly arising by contamination of the isolation cabinet in which the mice were kept, was responsible for induction of at least the majority of the tumours (Gaugas *et al.*, 1969). Anti-polyoma antibodies were ultimately present in the normal control mice, so these must have been similarly exposed to the virus but remained tumour free. In fact, polyoma virus is commonly present in many laboratory and wild mouse colonies but tumours are rarely, if ever, seen (Rowe, Hartley and Huebner, 1961). In contrast, inoculation of the virus into newborn mice and certain other rodents, carnivores and lagomorphs is followed by the formation of a variety of lesions in different tissues (Stewart, Eddy and Borgese, 1958; Chesterman, 1961; Stanton and Otsuka, 1963; Pomerance and Chesterman, 1965; Lehman and Defendi, 1970).

Since reporting our findings, we again attempted to propagate human leprosy bacilli in immunosuppressed mice. The appearance of polyoma-type tumours in ALG-treated mice again curtailed the study. In contrast, mice which had been kept under similar conditions after adult thymectomy and irradiation (900 rad) showed only a very low incidence of tumours. Investigations were therefore carried out to obtain information on immunological protection against incidental and deliberate infection with polyoma virus in normal, immunosuppressed (adult thymectomy combined with either ALG or irradiation) and in immune reconstituted mice.

### MATERIALS AND METHODS

*Immunosuppression of mice.*—Female virgin CBA mice (inbred conventional colony) were thymectomized surgically when 6 weeks old. One and 14 days after thymectomy those mice about to receive antilymphocytic globulin (ALG) were " primed " by giving an intraperitoneal injection of 0·1 ml of normal rabbit globulin (NRG); this increases the effectiveness of subsequent ALG administration, presumably because mice become tolerant to rabbit globulin (Lance and Dresser, 1967; Gaugas and Rees, 1968). Alternatively, 2–3 weeks after thymectomy the mice were exposed to 900 rad whole-body irradiation and their haemopoietic tissues were reconstituted by transplantation of isogeneic bone marrow cells (Gaugas *et al.*, 1969). Mice in another group were neonatally thymectomized (*i.e.* within 36 hours of birth). Completeness of the operations was confirmed at autopsy.

*Preparation of antilymphocytic globulin.*—Anti-mouse lymphocytic serum (ALS) was raised in New Zealand white rabbits (2–3 kg body weight) by injection of thymocytes collected from 6-week-old CBA mice (Levey and Medawar, 1966) in a suspension freed of contaminating erythrocytes (Boyle, 1968); after complement inactivation (heating at 56° for 30 min) erythrocytotoxic antibody was absorbed by addition of isologous erythrocytes (approximately 5% packed cells added for 30 min at 37°). The globulin fraction (ALG) was prepared by precipitation with ammonium sulphate solution which was later removed by dialysis (Stelos, 1967). Immunosuppressive potency of the ALG was demonstrated using the skin allograft reaction across an H-2 locus barrier in a standard procedure (Levey and Medawar, 1966). Mice were injected subcutaneously into the interscapular region once weekly with ALG equivalent to 0·4 ml of ALS. Mice were housed 6 to a metal cage (groups of 14–24 mice) and kept in an isolation cabinet. Sterile disposable syringes and needles were used to administer sera, and were renewed after injecting the mice housed in each cage.

*Leprosy infection.*—Mice, 8–9 weeks old, were infected by inoculation of $10^5$ *M. leprae* (in sterile 0·1% bovine albumin–saline) into both hind footpads. Bacilli were obtained by homogenization of a leproma freshly excised from an untreated patient.

*Polyoma virus infection.*—Mice were infected by intraperitoneal injection of $10^5$ TCD polyoma virus (Mill Hill strain) and were housed in a separate building and not inoculated with *M. leprae.*

*Polyoma haemagglutination-inhibition (HI) antibody test.*—After treatment with receptor-destroying enzyme (Wellcome), mouse sera were titrated for polyoma HI antibodies according to standard techniques (Rowe *et al.*, 1959; Hartley and Rowe, 1959). Stock mice were found to be free of detectable polyoma HI antibodies at the beginning of the experiments.

*Adoptive transfer of immunity against polyoma tumours.*—Adult CBA mice were injected with polyoma virus and after 3 weeks the spleens were excised. Spleen cell suspensions were prepared (in sterile balanced salt solution) and were injected either intraperitoneally or intravenously so that each isogeneic recipient received $2 \times 10^7$ cells. Control mice were injected with normal spleen cells.

*Polyoma antibody.*—Adult CBA mice of both sexes were infected with polyoma virus and 3 weeks later they were bled out and serum taken. A pool of antibody with a HI titre of 2460 and a neutralization titre of greater than 100 was obtained. Each recipient was inoculated intraperitoneally with 0·2 ml of this pool.

*Autopsy of mice.*—Moribund tumour-bearing mice were killed by cervical dislocation and full autopsies were performed. Tissues (tumour, intestines, spleen, adrenal gland, liver, kidney, lung, heart, salivary glands and lymph nodes) were fixed in formol–acetic–alcohol; bony tissues were decalcified in versene. After embedding in paraffin wax, tissues were sectioned at 4 $\mu$m and stained with Ehrlich's haematoxylin and eosin, carbol fuchsin for acid–alcohol fast bacilli, or PAS for fungi.

### RESULTS

### *Incidental infection with polyoma virus in immunosuppressed mice*

Mice treated by thymectomy, either alone or combined with ALS administration or 900 rad irradiation, were observed throughout their lifespan for the appearance of tumours or other pathological abnormality. Malignant tumours, and occasionally non-malignant tumours, began to appear in thymectomized mice 4 months after commencement of ALG treatment. The frequency and distribution of tumours amongst the various groups of experimental mice are shown in Table I (Group A). When tumours first began to appear in these thymectomized–ALG-treated mice, half the number of remaining mice of this group which had no detectable tumours (subsequently called Group A1) received an intraperitoneal injection of $2 \cdot 0 \times 10^7$ viable splenic mononuclear cells collected from adult CBA mice that had previously been immunized against polyoma virus (*i.e.* adoptive immunization of the immunosuppressed mice). These mice received no further treatment with ALG. Only in the Group B mice (Table I) was ALG treatment continued until the mice became moribund. Over a period of 24 months tumours developed in all 17 mice not receiving spleen cells, whereas none of the 14 mice with adoptive immunity developed tumours (Table I). The distribution, frequency and classification of tumours and other abnormalities are listed in Table II.

TABLE. 1—*Tumours Appearing in Immunosuppressed Female CBA Mice (Observed for up to Two Years)*

| Group | Thymectomy at 6 weeks | Primed at 6 and 8 weeks | ALG | 900 rad | No. mice with tumours/ no. treated |
|---|---|---|---|---|---|
| A | + | + | + | − | 17/17 |
| A1* | + | + | + | − | 0/14 |
| B | − | + | + | − | 22/24† |
| C | + | − | − | + | 1/18 |
| D (controls) | − | − | − | − | 0/18 |
| E | Neonatal thymectomy | − | − | − | 0/16 |

* Group A1 with adoptive immunity (see text for details).
† Two mice died with non-malignant granulomata.
All mice were infected with leprosy except those in group E.

Another group of 24 mice received ALG alone without prior thymectomy (Group B); 22 of these eventually developed tumours, which first appeared 7·5 months after commencement of ALG administration. The 2 remaining mice of this group died with extensive non-malignant granulomata, possibly caused by fungi since fungal elements were sometimes found in the mouse tissues. Animals in this group developed tumours after a longer interval than those observed in thymectomized mice treated with ALG (Group A). Somewhat surprisingly, only one mouse in the thymectomized–900 rad irradiated group (Group C) developed a tumour, an osteosarcoma.

Tumours were of various histological types, mostly rapidly growing adenocarcinomata, probably arising in breast; osteosarcomata (in ribs, vertebrae, pelvic girdle or tibia), and fibrosarcomata, possibly arising in the rib periosteum. Unlike in our previous study (Gaugas *et al.*, 1969), salivary gland tumours—which are the most common type of polyoma tumour in most mouse strains infected with most strains of the virus—were rarely found. As expected, bone tumours grew much less rapidly than adenocarcinomata. No reticulo-endothelial tumours were obtained but the lymph nodes of mice that had received ALG showed the features characteristic of such treatment, namely involution of the paracortical regions and replacement with macrophages and filling of the medulla with plasma cells. Tumours failed to appear in normal

TABLE II.—*Time of Appearance, Site and Classification of Tumours and Other Lesions in Immunosuppressed Female CBA Mice*

| Group* | Appearance after starting ALG or 900 rad treatments (months) | Site of tumour or other lesion | Numbers and classification |
|---|---|---|---|
| A | 4–10 | Subcutaneous | 4 Undifferentiated |
| | | | 1 Breast adenocarcinoma |
| | | | 6 Poorly differentiated adenocarcinomata |
| | | | 1 Undifferentiated spindle-cell sarcoma |
| | | | 1 "Carcino-sarcoma " type |
| | | Pelvic girdle, rib, vertebrae or tibia | 5 Osteosarcomata |
| | | Uterus | 1 Sarcoma |
| | | Salivary gland | 1 Sarcoma |
| | | Xiphisternum, skin | 2 Haemangiomata |
| | | Stomach | 2 Non-malignant ulcers |
| B† | 7½–15 | Subcutaneous | 8 Undifferentiated |
| | | | 1 Breast adenocarcinoma |
| | | | 9 Poorly differentiated adenocarcinomata |
| | | | 2 Spindle-cell sarcomata |
| | | | 1 Epithelial tumour |
| | | | 1 Fungal granuloma |
| | | Pelvic girdle, vertebrae, 7 ribs | 7 Osteosarcomata |
| | | | 1 Peri-osteal sarcoma |
| | | Salivary gland | 1 Sarcoma |
| | | Adrenal gland | 1 Polygon-cell carcinoma |
| | | Lymph node | 1 Secondary carcinoma |
| | | Lungs | 1 Secondary carcinoma |
| | | | 1 Emboli of tumour cells in vessels‡ |
| | | Liver | 1 Paraportal collection of pigment cells |
| | | | 1 Calcified small lesion |
| C | 5 | Bone | 1 Osteosarcoma |

* Groups D (controls) and E (neonatally thymectomized mice) showed no tumours.
† Fluid-filed ovarian cysts were seen in 2 Group B mice, but were not examined histologically.
‡ Mouse also had an undifferentiated subcutaneous tumour.

control mice, which were observed for 2 years. No tumours appeared in a group of 16 neonatally thymectomized mice observed until their deaths after 1·5–6 months.

Adenocarcinomata, which were solid in immunosuppressed mice, were transplantable under the skin of normal CBA mice. In this situation they quickly showed central necrosis and much infiltration by lymphocytes, macrophages and some polymorphonuclear leucocytes. The tumours then lost their original solid appearance, but remained malignant, infiltrating into muscle and retaining their transplantability. This histological picture indicates that the tumour in the normal mouse elicited a cell-mediated reaction, with possibly a mild antibody-mediated response superimposed.

A control group of mice injected twice weekly, from the day of birth with 0·1–0·5 ml of normal rabbit serum until 6 weeks old, failed to develop tumours in their lifespan (12 mice). This result suggests that polyoma virus was absent from the rabbit serum used.

Recently, Nehlsen (1971) treated mice throughout their lifespan with heterologous antithymocyte serum and obtained a

FIG. 1.—Mammary adenocarcinoma. × 230.

FIG. 2.—Secondary tumour in lung. × 230.

FIG. 3.—Osteosarcoma. × 230.

high incidence of amyloid in the mice (Simpson and Nehlsen, 1971). Only a few of our mice showed amyloid in the spleen and kidney, which may have been due to the fact that we used ALG rather than ALS. The amyloid in Nehlsen's mice may have been induced by the higher serum protein content (in particular albumin) in the ALS used.

All groups of mice had HI antibody titres ranging from 320 to 12,800 when tested towards the end of the experiment, showing that they had been infected with polyoma virus.

*Deliberate infection of immunosuppressed mice with polyoma virus*

Virgin female mice of the CBA strain were thymectomized at 6 weeks and given weekly subcutaneous injections of 0·4 ml rabbit antilymphocytic globulin (ALG). Control mice were given normal rabbit globulin (NRG). Ten days after the inception of ALG or NRG treatment, mice were given intraperitoneal injections of $10^5$ $TDC_{50}$ polyoma virus. ALG treatment was continued for 7 weeks, at which time the first tumour appeared. At the eighth week the mice were divided into 3 groups: one was untreated, the second was given spleen cells from normal adult CBA mice ($10^6$ cells per g bodyweight of recipient intravenously), and the third was given the same number of spleen cells from adult CBA mice infected 3 weeks previously with polyoma virus. No further ALG or NRG was given.

The results are shown in Table III. Before infection, none of the mice had antibodies against polyoma virus. All sera tested at 8 weeks in all groups showed haemagglutination inhibiting antibodies against polyoma virus. All of the thymectomized and ALG-treated mice with no restoration, and all but one of the mice restored with normal lymphoid cells,

TABLE III.—*Development of Tumours in Mice Infected with Polyoma Virus in the Absence of Leprosy*

| Preliminary treatment | Restoration at 7 weeks | No. of animals | % with tumours |
|---|---|---|---|
| NRG | None | 24 | 0 |
| Thymectomy + ALG | None | 14 | 100 |
| Thymectomy + ALG | Normal lymphoid cells | 10 | 90 |
| Thymectomy + ALG | Sensitized lymphoid cells | 11 | 0 |

showed tumours. None of the mice receiving sensitized lymphoid cells has developed a tumour during 18 months of further observation.

To examine further the effects of restoration of cell-mediated immunity, 18 mice with small mammary tumours about 5 mm in diameter were taken and the tumours excised under ether anaesthesia. Half of the mice were given $5 \times 10^6$ polyoma-sensitized syngeneic spleen cells intravenously, and the others left alone. All the mice in the latter group developed tumours—due to recurrence or separate origin; only 1 of the 9 mice receiving sensitized lymphoid cells has developed a tumour.

In a similar experiment, thymectomy and ALG treatment was again found to give rise to tumours in all 14 adult CBA mice infected with polyoma virus. Administration of 0·2 ml polyoma antibody one day before virus infection prevented the occurrence of tumours in 10 animals, whereas administration of polyoma antibody at 7 and 8 weeks had no demonstrable effect on the appearance of tumours in 11 animals.

### DISCUSSION

These findings confirm the observation that thymectomy plus ALG treatment is a more potent immunosuppressive regimen for induction of polyoma tumours than either procedure alone, and is much more effective than thymectomy plus 900 rad whole-body irradiation. Law (1966) and Allison and Taylor (1967) found tumours in only a minority of thymectomized mice infected as adults with polyoma virus and Nehlsen (1971) had similar results after administration of ALS alone.

Viruses other than polyoma, for example the mammary tumour agent or FBJ virus (Finkel, Biskis and Jinkins, 1966), might conceivably have been activated by the immunosuppressive treatments, although no evidence of concurrent infection with other viruses was found in this or in our previous studies (Gaugas *et al.*, 1969). Nevertheless, a few mice succumbed to a fungal infection which was not specifically identified. Thus an enhancement in susceptibility to fungal infection as well as virus oncogenesis were operative. The prevention of tumours by transfers of lymphoid cells from polyoma-immune animals provides further evidence that the spontaneously appearing tumours in our mice infected with leprosy were in fact induced by the virus.

Local persistence and proliferation of *M. leprae* was probably insufficient to have exerted any immunosuppressive effect (Turk and Waters, 1968; Ptak *et al.*, 1970), since no macroscopical leprosy lesions were obtained in the injected footpads. Indeed, the relatively few bacilli present may have had an adjuvant effect on the production of immunity to whatever degree it existed in the immunosuppressed mice throughout their lifespan. However, the fact that similar results were obtained in the absence of leprosy infection shows that the organisms were unnecessary.

Taken together, observations from both the incidental and deliberate polyoma virus infection experiments demonstrate that the main factor allowing virus oncogenesis in the thymectomized–ALG-

treated adult mouse is suppression of cell-mediated immunity. The effect can be reversed by restoration with lymphoid cells from specifically sensitized syngeneic donors, even when these were carried out long after infection and, presumably, oncogenic transformation of cells in the recipients. The failure of restoration by normal lymphoid cells is presumably a result of timing and parallels previous experience of restoration after neonatal thymectomy (Allison, 1970): the normal lymphoid cells prevent tumour formation only when transferred one week after virus inoculation, whereas sensitized cells are effective when transferred one month after virus inoculation. The prevention of reappearance of excised tumours by transfer of sensitized lymphoid cells shows that under optimal conditions immunotherapy can be a remarkably effective adjunct to surgery.

The results presented in this paper provide the strongest experimental evidence yet available for the existence of an immunological surveillance mechanism against oncogenesis. Many mouse colonies carry polyoma virus, which is potentially oncogenic, and yet natural infections hardly ever result in tumours. In such colonies there is no vertical transmission of virus. Newborn mice are passively protected by maternal antibody, and are infected only when this passive protection has waned. By this age their immune responses against polyoma tumours are early and effective (Allison, 1970). There is, however, no limitation of the capacity of the virus to multiply and produce an oncogenic transformation in host cells, as the results of infecting highly immunosuppressed animals show.

### REFERENCES

ALLISON, A. C. (1970) On the Absence of Tolerance in Virus Oncogenesis. In *Proc. Fourth Quadrennial Cancer Conference, Perugia*, 1969. Ed. L. Severi. Division of Cancer Research. p. 563.

ALLISON, A. C. & LAW, L. W. (1968) Effects of Antilymphocytic Serum on Virus Oncogenesis. *Proc. Soc. exp. Biol. Med.*, **127**, 207.

ALLISON, A. C. & TAYLOR, R. B. (1967) Observations on Thymectomy and Carcinogenesis. *Cancer Res.*, **27**, 703.


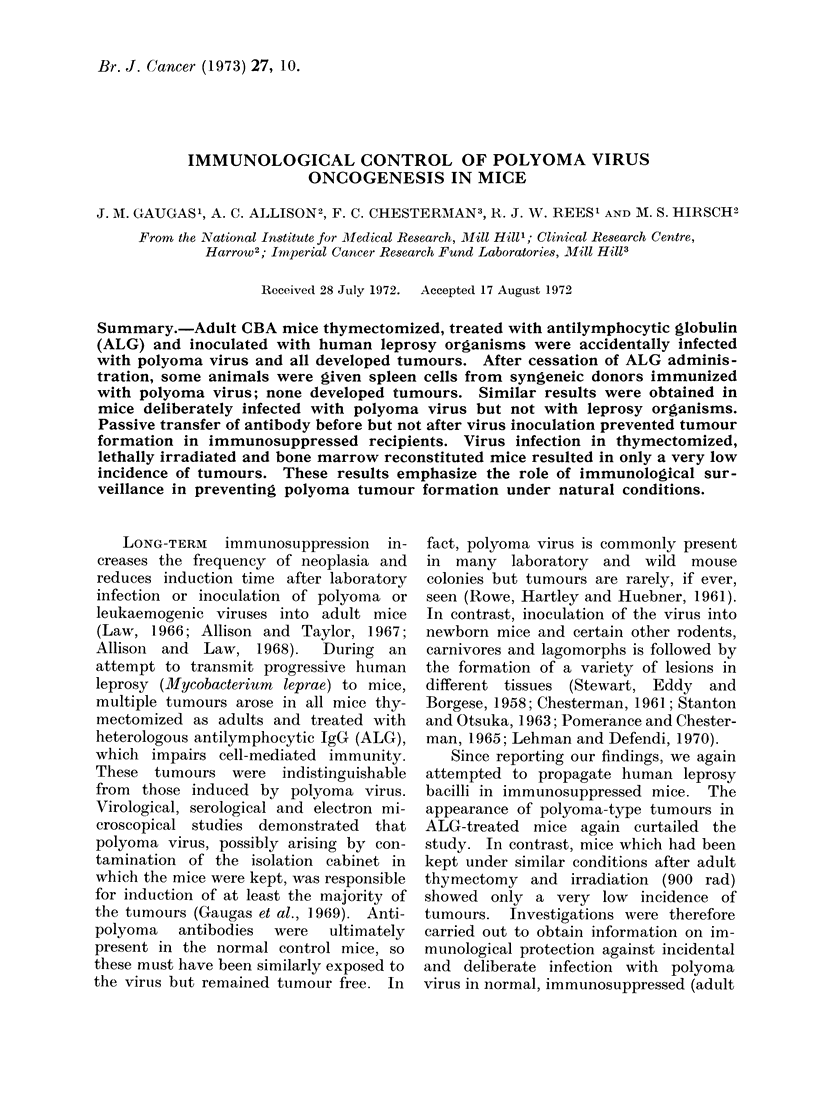

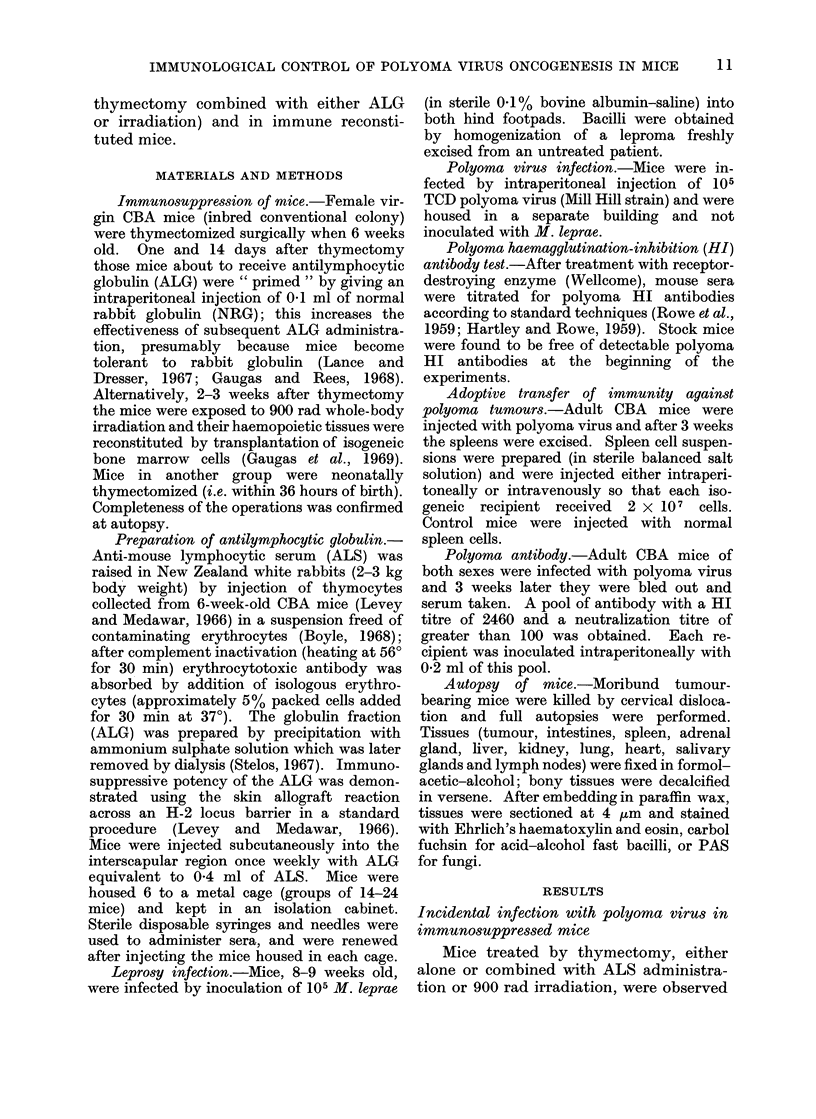

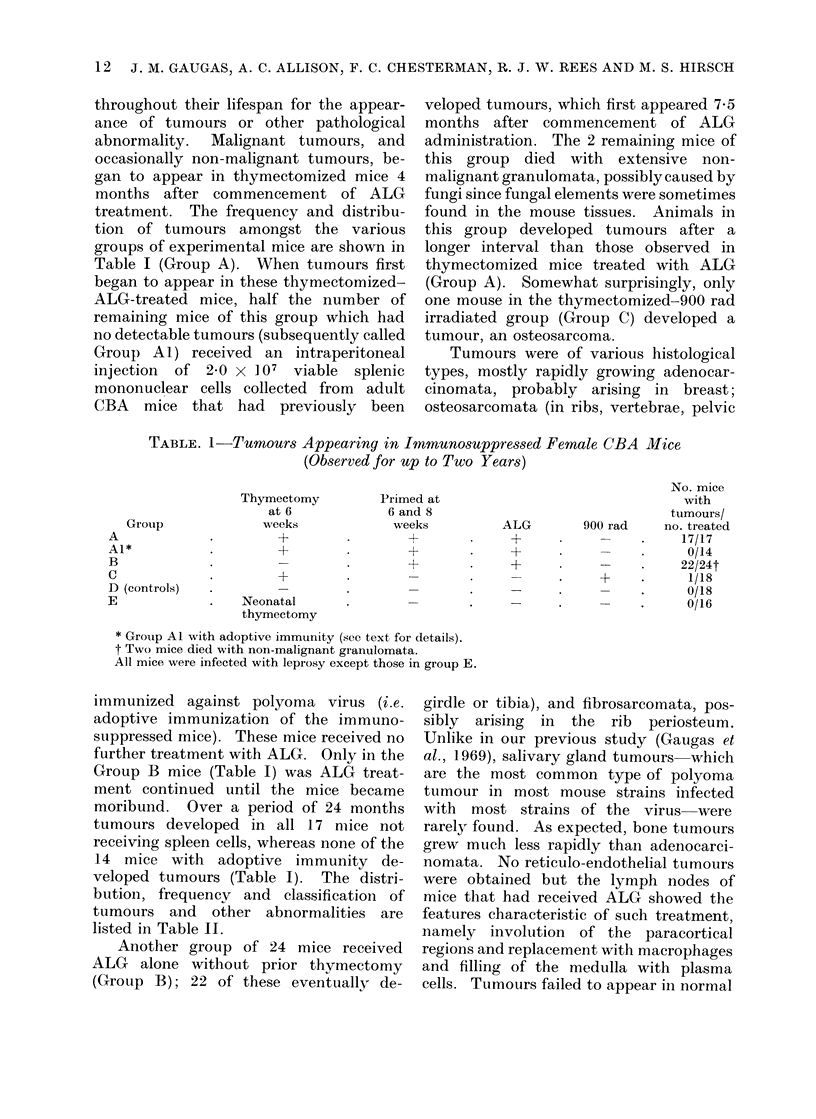

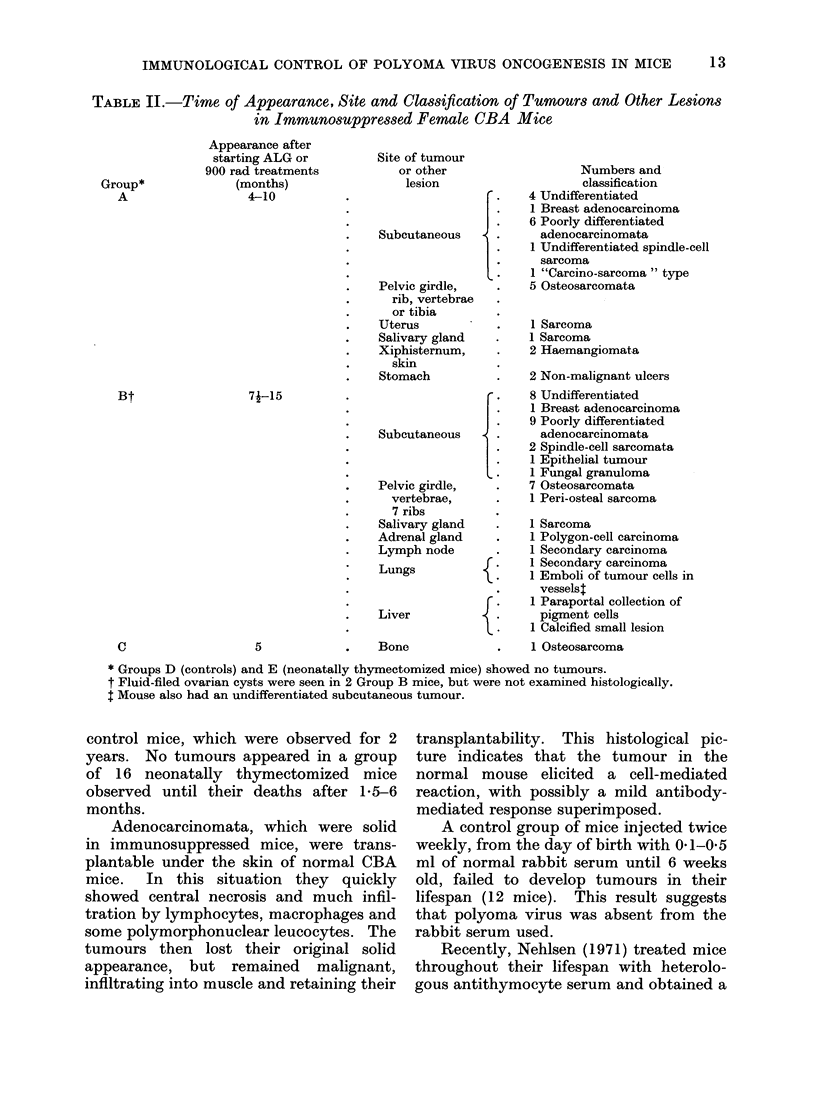

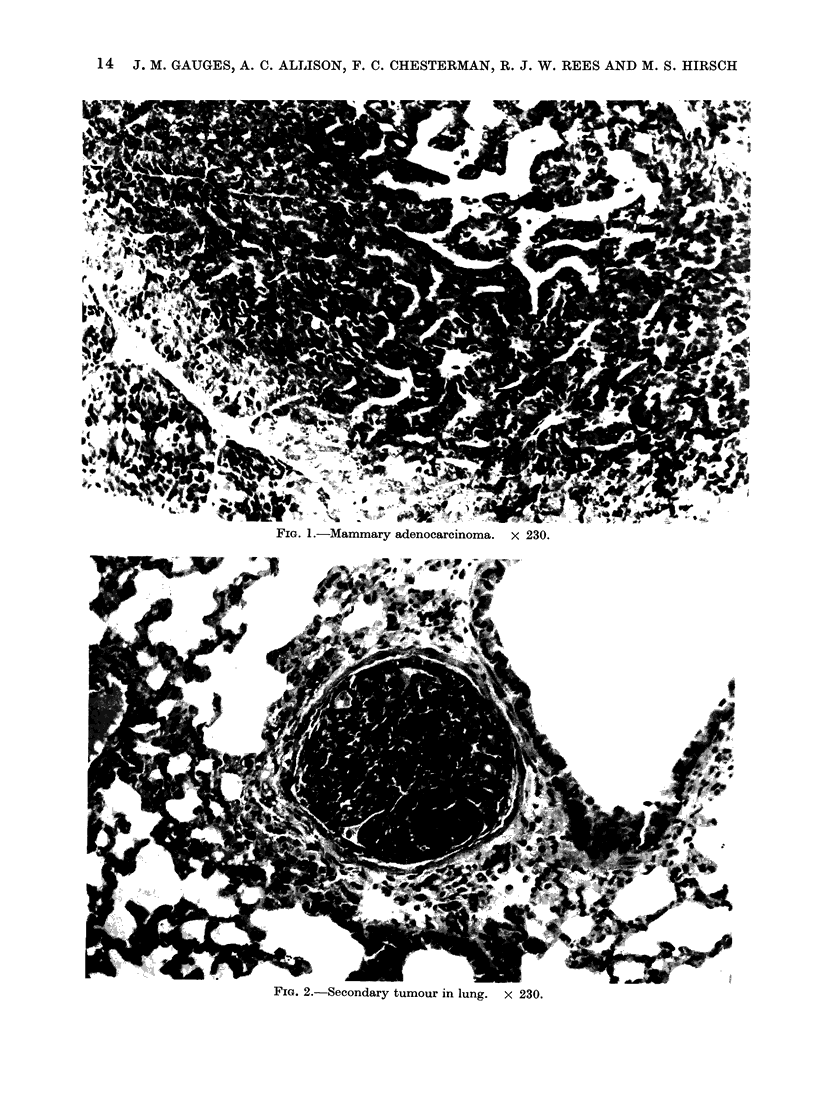

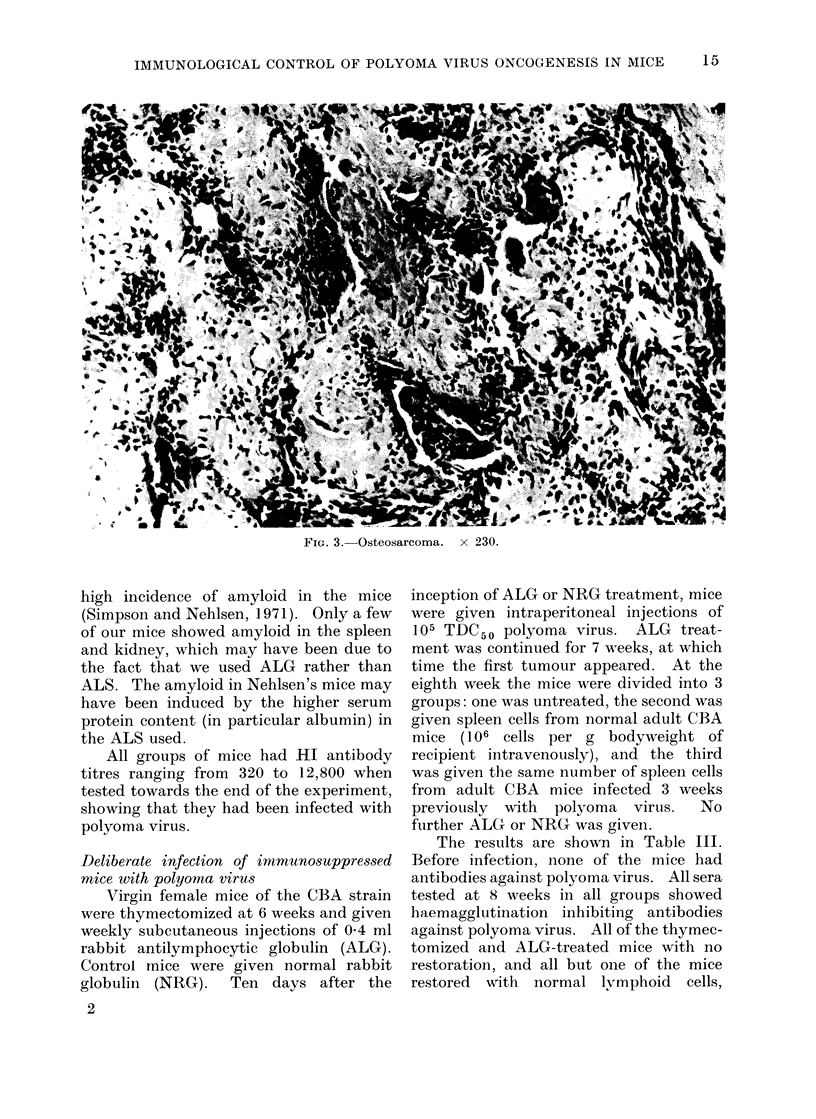

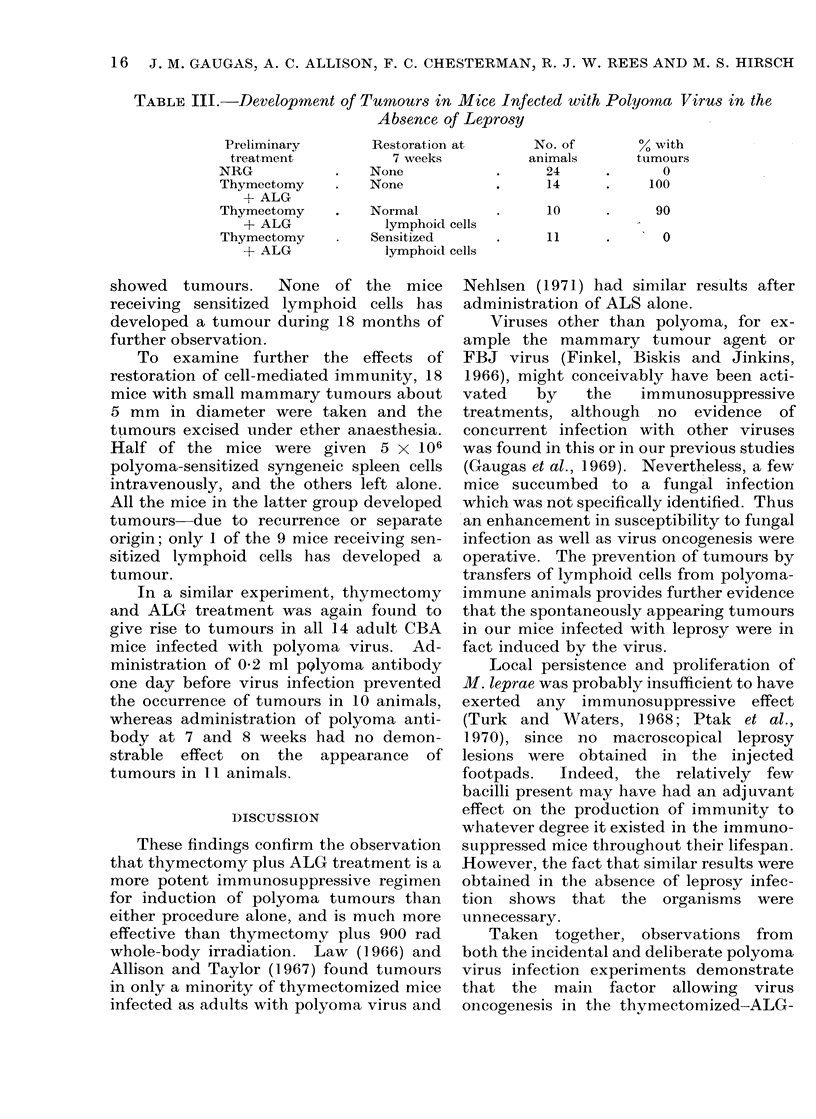

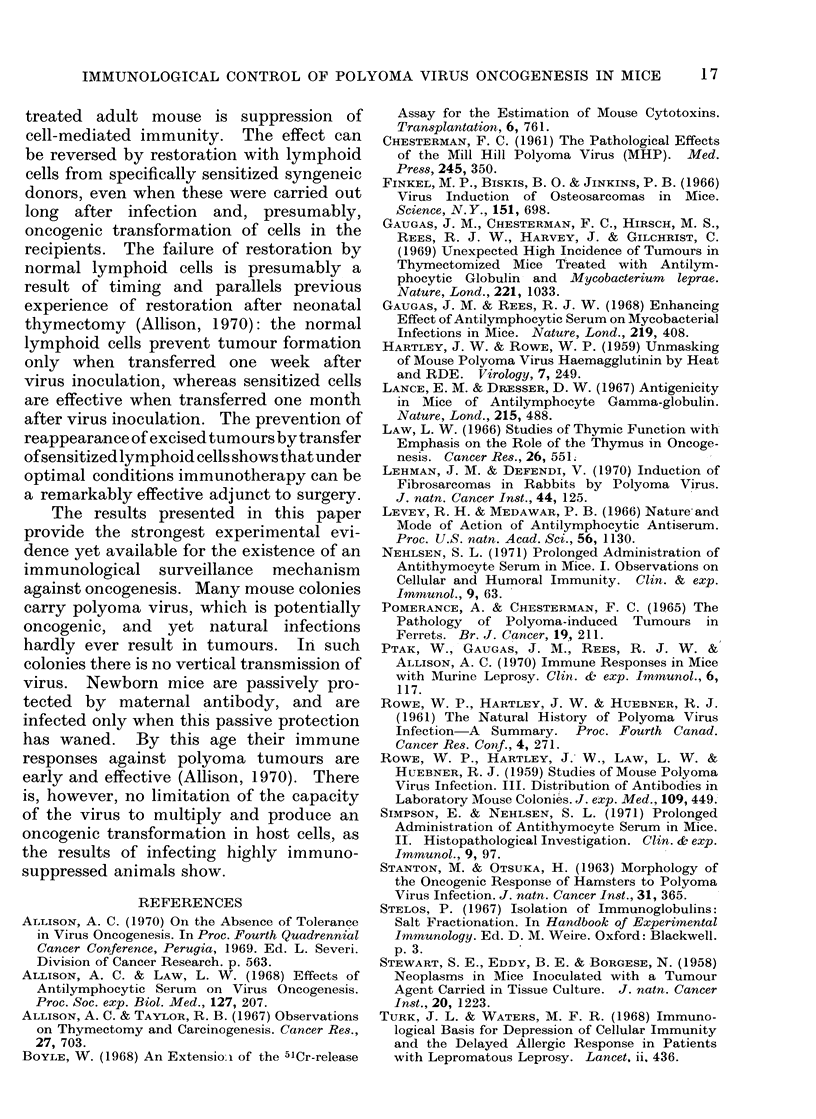


## References

[OCR_00773] Allison A. C., Law L. W. (1968). Effects of antilymphocyte serum on virus oncogenesis.. Proc Soc Exp Biol Med.

[OCR_00778] Allison A. C., Taylor R. B. (1967). Observations on thymectomy and carcinogenesis.. Cancer Res.

